# Machine Teaching for Human Inverse Reinforcement Learning

**DOI:** 10.3389/frobt.2021.693050

**Published:** 2021-06-30

**Authors:** Michael S. Lee, Henny Admoni, Reid Simmons

**Affiliations:** Robotics Institute, Carnegie Mellon University, Pittsburgh, PA, United States

**Keywords:** inverse reinforcement learning, learning from demonstration, scaffolding, policy summarization, machine teaching

## Abstract

As robots continue to acquire useful skills, their ability to teach their expertise will provide humans the two-fold benefit of learning from robots and collaborating fluently with them. For example, robot tutors could teach handwriting to individual students and delivery robots could convey their navigation conventions to better coordinate with nearby human workers. Because humans naturally communicate their behaviors through selective demonstrations, and comprehend others’ through reasoning that resembles inverse reinforcement learning (IRL), we propose a method of teaching humans based on demonstrations that are informative for IRL. But unlike prior work that optimizes solely for IRL, this paper incorporates various human teaching strategies (e.g. scaffolding, simplicity, pattern discovery, and testing) to better accommodate human learners. We assess our method with user studies and find that our measure of test difficulty corresponds well with human performance and confidence, and also find that favoring simplicity and pattern discovery increases human performance on difficult tests. However, we did not find a strong effect for our method of scaffolding, revealing shortcomings that indicate clear directions for future work.

## 1 Introduction

As robots become capable in tasks once accomplished only by humans, the extent of their influence will depend in part on their ability to teach and convey their skills. From the youngest of us learning to handwrite (Sandygulova et al., 2020; Guneysu Ozgur et al., 2020) to practitioners of crafts such as chess, many of us stand to benefit from robots that can effectively teach their mastered skill. Furthermore, our ability to collaborate fluently with robots partly depends our understanding of their behaviors. For example, workers at a construction site could better coordinate with a new delivery robot if the robot could clearly convey its navigation conventions (e.g. when it would choose to go through mud over taking a long detour).

While demonstrations are a natural method of teaching and learning behaviors for humans, its effectiveness still hinges on conveying an informative set of demonstrations. The literature on how humans generate and understand behaviors provides insight into what makes a demonstration informative. Cognitive science suggests that humans often model one another’s behavior as exactly or approximately maximizing a reward function (Jern et al., 2017; Jara-Ettinger et al., 2016; Lucas et al., 2014), which they can infer through reasoning resembling inverse reinforcement learning (IRL) (Ng and Russell, 2000; Jara-Ettinger, 2019; Baker et al., 2009; Baker et al., 2011). Furthermore, humans are often able to obtain a behavior that (approximately) maximizes a reward function through planning, which can be modeled as dynamic programming or Monte Carlo tree search (Shteingart and Loewenstein, 2014; Wunderlich et al., 2012).

Putting these insights together, we can often expect humans to be able to model others’ behaviors once equipped with their reward functions.^1^ For example, upon seeing a new worker consistently arrive on time to each workday, a manager will infer that the worker places high values on punctuality and consistency and will arrive promptly at other work-related functions. Thus, the problem of conveying a behavior or skill can be reduced to conveying the underlying reward function, and the informativeness of a demonstration can be quantified by how much information it reveals regarding the reward function using IRL.

Though IRL offers a principled measure of a demonstration’s informativeness, human learning is multi-faceted and is also influenced by other factors, such as the simplicity of explanations (Lombrozo, 2016). Thus, unlike prior work on machine teaching that optimizes solely for IRL (Brown and Niekum, 2019), this paper incorporates insights on how humans effectively learn to further accommodate human learners.

In this work, we explore whether augmenting IRL with insights from human teaching improves human learning over optimizing for IRL alone. We first employ *scaffolding* from social constructivism (learning theory) to encourage demonstrations that are not just informative but also comprehensible. Specifically, we assume a general human learner without prior knowledge, and sequence demonstrations that incrementally increase in informativeness and difficulty. Noting the cognitive science literature that suggests humans favor simple explanations that follow a discernible pattern (Lombrozo, 2016; Williams et al., 2010), we also optimize for visual *simplicity and pattern discovery* when selecting demonstrations. Finally, toward effective *testing* of the learner’s understanding, we show that the measure of a demonstration’s informativeness during teaching can be inverted into a measure of expected difficulty for a human to predict that exact demonstration during testing.

Two user studies strongly correlate our measure of test difficulty with human performance and confidence, with low, medium, and high difficulty tests yielding high, medium, and low performance and confidence respectively. Study results also show that favoring simplicity and pattern discovery significantly increases human performance on difficult tests. However, we do not find a strong effect for our method of scaffolding, revealing shortcomings that indicate clear directions for future work.

## 2 Related Work

### 2.1 Policy Summarization and Machine Teaching

The problem of policy summarization considers which states and actions should be conveyed to help a user obtain a global understanding of a robot’s policy (i.e. behavior or skill) (Amir et al., 2019). There are two primary approaches to this problem. The first relies on heuristics to evaluate the value of communicating certain states and actions, such as entropy (Huang et al., 2018), differences in Q-values (Amir and Amir, 2018), and differences between the policies of two agents (Amitai and Amir, 2021).

We build on the second approach, which follows the machine teaching paradigm (Zhu et al., 2018). Given an assumed learning model of the student (e.g. IRL to learn a reward function), the machine teaching objective is to select the minimal set of teaching examples (i.e. demonstrations) that will help the learner arrive at a specific target model (e.g. a policy). Though machine teaching was first applied to classification and regression (Zhu, 2015; Liu and Zhu, 2016), it has also recently been employed to convey reward functions from which the corresponding policy can be reconstructed. Huang et al. (2019) selected informative demonstrations for humans modeled to employ approximate Bayesian IRL for recovering the reward. This technique requires the true reward function to be within a candidate set of reward functions over which to perform Bayesian inference, and computation scales linearly with the size of the set. Cakmak and Lopes (2012) instead focused on IRL learners and selected demonstrations that maximally reduced uncertainty over all viable reward parameters, posed as a volume removal problem. Brown and Niekum (2019) improved this method (particularly for high dimensions) by solving an equivalent set cover problem instead with their Set Cover Optimal Teaching (SCOT) algorithm. However, SCOT is not explicitly designed for human learners and this paper builds on SCOT to address that gap.

### 2.2 Techniques for Human Teaching

Human teaching and learning is a multifaceted process that has been studied extensively. Thus, we also take inspiration from social constructivism (learning theory) and cognitive science in informing how a robot may teach a skill to a human learner so that the learner may correctly reproduce that skill in new situations.

**Scaffolding**: Scaffolding is a well-established pedagogical technique in which a more knowledgeable teacher assists a learner in accomplishing a task currently beyond the learner’s abilities, e.g. by reducing the degrees of freedom of the problem and/or by demonstrating partial solutions to the task (Wood et al., 1976). Noting the benefits seen by automated scaffolding to date [e.g. Sampayo-Vargas et al. (2013)], we implement the first recommendation made by Reiser (2004) for software-based scaffolding, which is to reduce the complexity of the learning problem through additional structure. Specifically, we incorporate this technique when teaching a skill by providing demonstrations that sequentially increase in informativeness and difficulty.

**Simplicity and Pattern Discovery**: Studies on explanations preferred by humans indicate a bias toward those that are simpler and have fewer causes (Lombrozo, 2016). Furthermore, Williams et al. (2010) found that explanations can be detrimental if they do not help the learner to notice useful patterns or even mislead them with false patterns. Together, these two works support the idea that explanations should minimize distractions that potentially inspire false correlations and instead highlight and reinforce the minimal set of causes. We thus also optimize for simplicity and pattern discovery when selecting demonstrations that naturally “explain” the underlying skill.

**Testing**: Effective scaffolding requires an accurate diagnosis of the learner’s current abilities to provide the appropriate level of assistance throughout the teaching process (Collins et al., 1988). A common diagnostic method is presenting the learner with tests of varying difficulties and assessing their understanding of a skill. Toward this, we propose a way to quantify the difficulty of a test that specifically assesses the student’s ability to predict the right behavior in a new situation.

## 3 Technical Background

### 3.1 Markov Decision Process

The robot’s environment is represented as an instance (indexed by *i*) of a deterministic^2^ Markov decision process, $MDP_{i}:=\left(S_{i},A,T_{i},R,\gamma ,S_{i}^{0}\right)$, where $S_{i}$ and A denote the state and action sets, $T_{i}:S_{i}\times A\to S_{i}$ the transition function, R:S×A→ℝ the reward function, γ∈[0,1] the discount factor, and $S_{i}^{0}$ the initial state distribution, and $S:\cup_{i}S_{i}$ the union over the states of all related instances of MDPs, which we call a domain (to be described in the following paragraphs).

Finally, the robot has an optimal policy (i.e. a skill) $\pi^{\text{*}}{\;}_{i}:S_{i}\to A$ that maps each state in an MDP to the action that will optimize the reward in an infinite horizon. A sequence of $\left(s_{i},a,s^{\text{′}}{\;}_{i}\right)$ tuples obtain by following π* gives rise to an optimal trajectory (i.e. a demonstration) ξ*, where $s_{i},s^{\text{′}}{\;}_{i}\in S_{i},a\in A$. We assume that *R* can be expressed as a weighted linear combination of *l* reward features^3^ $\phi :S\times A\to {\mathbb{R}}^{l}$, i.e. $\begin{array}{l} R=w{*}^{⊤}\;\phi \;\left(s,a,s\prime \right) \end{array}$ (Abbeel and Ng, 2004). We also assume that the human is aware of all aspects of an MDP (including the reward features) but not the weights w*.

**FIGURE 1:**
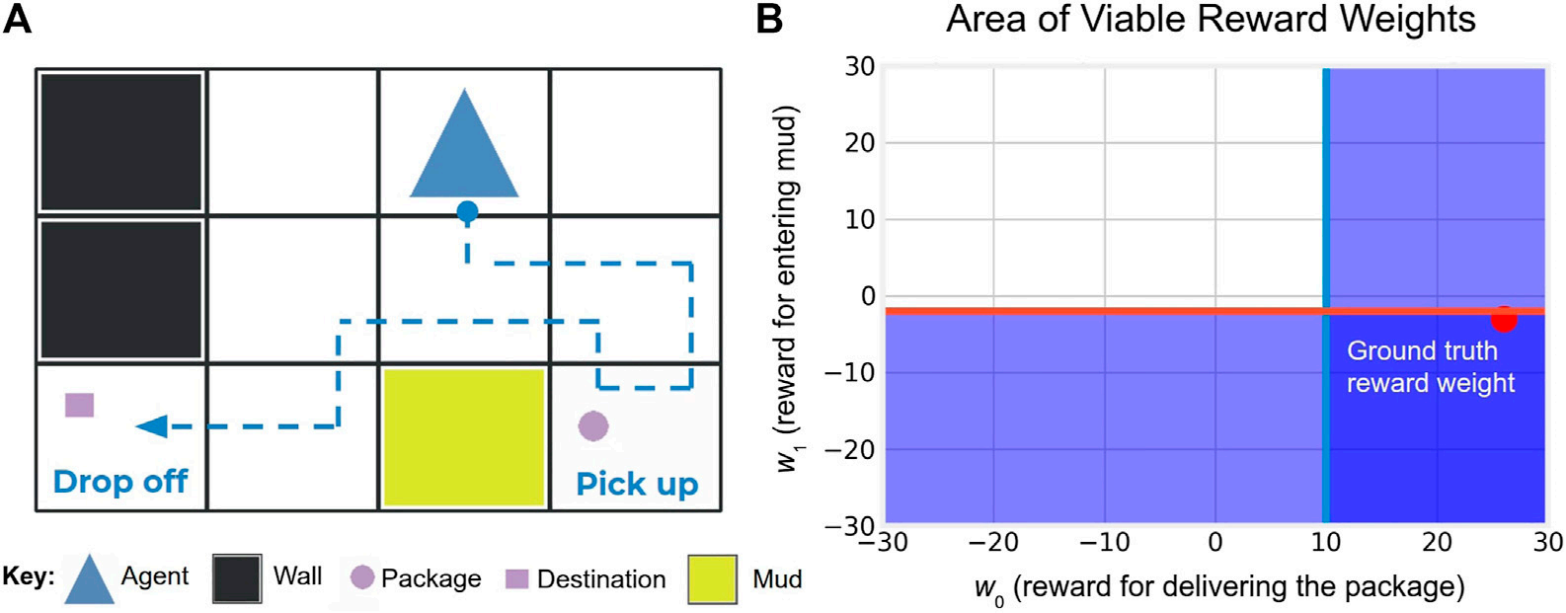
**(A)** A demonstration D of an optimal policy π in the delivery domain. Agent aims to deliver the package to the destination while avoiding walls and avoiding mud if the detour is not too costly. **(B)** The left demonstration can be translated into a set of half-space constraints on the underlying policy reward weights using Eq. 4. The darker shaded region is where all constraints (the red and light blue lines) hold true, which corresponds to the behavior equivalence class BEC(D|π), see Section 3.3.

**FIGURE 2:**
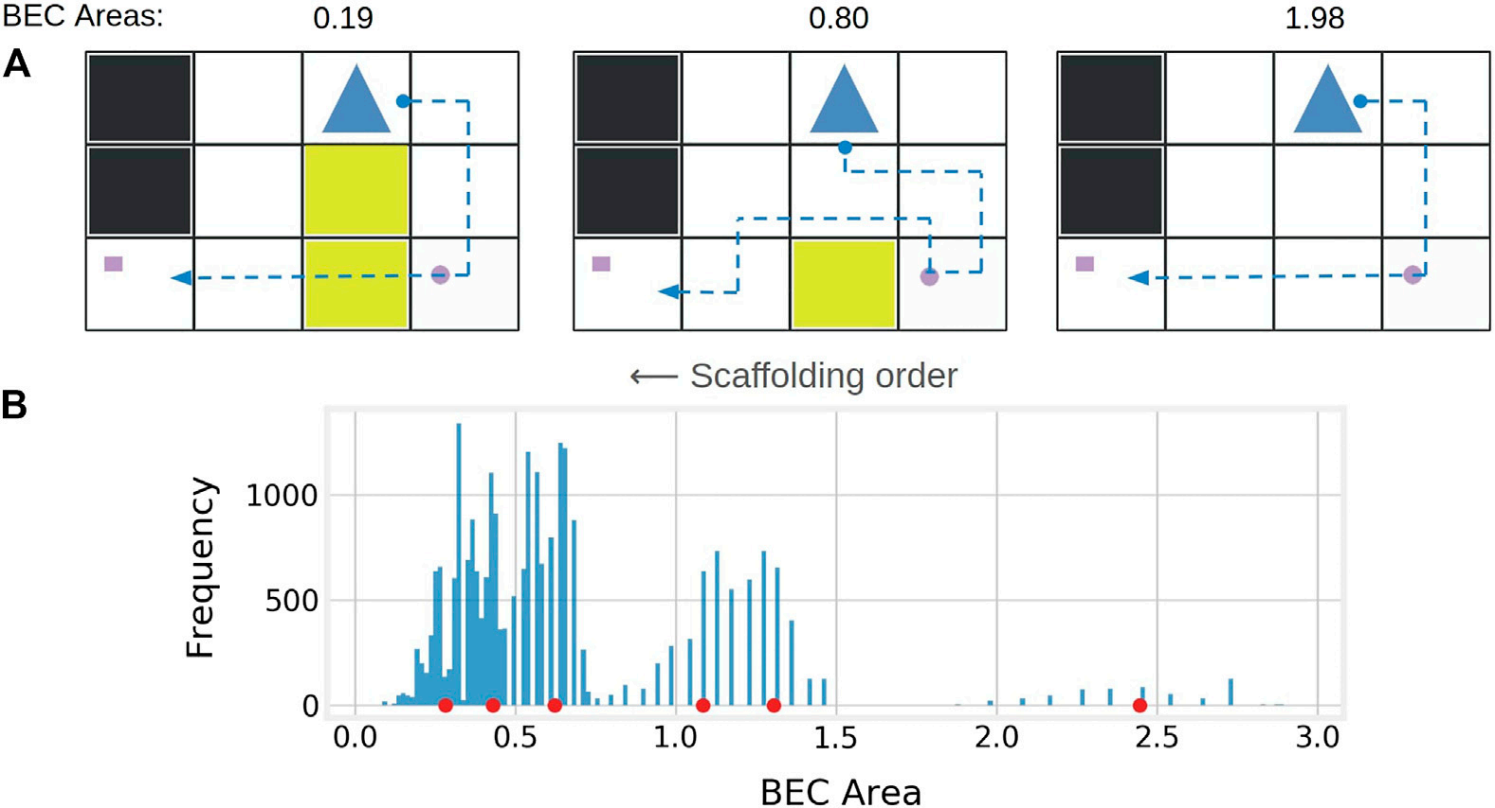
**(A)** Sample demonstrations exhibiting scaffolding, simplicity, and pattern discovery. We scaffold by showing demonstrations that incrementally decrease in BEC area (which appears to correlate inversely with informativeness and difficulty). Simplicity is encouraged by minimizing visual clutter (i.e. unnecessary mud patches). Pattern discovery is encouraged by holding the agent and passenger locations constant while highlighting the single additional toll between demonstrations that changes the optimal behavior. **(B)** Histogram of BEC areas of the 25,600 possible demonstrations in the delivery domain. Cluster centers returned by k-means (k = 6) are shown as red circles along the *x*-axis. Demonstrations from every other cluster are selected and shown in order of largest to smallest BEC area for scaffolded machine teaching.

Let a domain refer to a collection of related MDPs that share A,R,γ but differ in $S_{i},\;T_{i}$ and $S_{i}^{0}$. Take for example the delivery domain, which modifies the Taxi domain (Dietterich, 1998) by adding mud (see Figure 1). The robot is rewarded for efficiently delivering the package to the destination while avoiding the mud if the detour is not too costly. Though MDPs in this domain may vary in the number and locations of mud patches and subsequently offer a diverse set of demonstrations (e.g. see Figure 2), they importantly share the same reward function *R*.

Because instances of a domain share *R*, the various demonstrations all support inference over the same w* through IRL. Thus, we overload the notation π* to refer to any policy of a domain instance that optimizes a reward with w*. Furthermore, while a demonstration strictly consists of both an optimal trajectory ξ* (obtained by following π*) and the corresponding MDP (minus w*), we will refer to a demonstration only by ξ* in this work for notational simplicity.

Having represented the robot’s environment and policy, we now define the problem of generating demonstrations for teaching that policy through the lens of machine teaching.

### 3.2 Machine Teaching for Policies

As formalized by Lage et al. (2019), machine teaching for policies seeks to convey a set of demonstrations D of size *n* (i.e. the allotted budget for teaching set) that will maximize the similarity *ρ* between π* and the policy $\hat{\pi }$ recovered using a model ℳ on D

$$\underset{D\in \text{Ξ}}{\text{arg}\;\text{ max}}\rho \left(\hat{\pi }\left(D,ℳ\right),\pi \text{*}\right)\text{s}.\text{t}.\left|D\right|=n$$ (1)

where Ξ is the set of all optimal demonstrations of π* in a domain. We assume that the ℳ employed by humans to approximate the underlying w* is IRL. Once w* (and the subsequent reward function) is approximated, we assume that human learners are able to arrive at π*, i.e. the skill, through planning on the underlying MDP.

Thus, the teaching objective reduces to effectively conveying w* through well-selected demonstrations.^4^ In order to quantify the information a demonstration provides on w*, we leverage the idea of behavior equivalence classes.

### 3.3 Behavior Equivalence Class

The *behavior equivalence class* (BEC) of *π* is the set of (viable) reward weights under which *π* is still optimal. The larger the BEC(*π*) is, the greater the potential uncertainty over w* that is underlying the robot’s optimal policy.

$$\text{BEC}\left(\pi \right)=\left\{w\in {\mathbb{R}}^{l}|\pi \text{ optimal w}.\text{r}.\text{t}.\;R=w^{⊤}\phi \left(s,a,s\prime \right)\right\}$$ (2)

The BEC(*π*) can be calculated as the intersection of the following half-space constraints generated by the central IRL equation (Ng and Russell, 2000)

$$w^{⊤}\left(\mu_{\pi }^{\left(s,a\right)}-\mu_{\pi }^{\left(s,b\right)}\right)\geq 0\forall a\in \underset{a\prime \in A}{\text{arg}\;\text{max}}Q\text{*}\left(s,a\prime \right),b\in A,s\in S$$ (3)

where $\begin{array}{l} \mu_{\pi }^{\left(s,a\right)}\;=\;E\;\left[\sum_{t=0}^{\infty }\;\gamma^{t}\phi \left(s_{t}\right)\;|\pi ,s_{0}\;=\;s,a_{0}\;=\;a\right] \end{array}$ is the vector of expected reward feature counts accrued from taking action *a* in *s*, then following *π* after, and Q*(s,a) refers to the optimal Q-value in a state and a possible action (Watkins and Dayan, 1992).

Brown and Niekum (2019) proved that the BEC(D|π) of a set of demonstrations D of a policy *π* can be formulated similarly as the intersection of the following half-spaces

$$w^{⊤}\left(\mu_{\pi }^{\left(s,a\right)}-\mu_{\pi }^{\left(s,b\right)}\right)\geq 0,\forall \left(s,a\right)\in D,b\in A.$$ (4)

Using the Eq. 4, every demonstration can be translated into a set of constraints on the viable reward weights.

Consider an example in the delivery domain with A= {*up*, *down*, *left*, *right*, *pick up*, *drop*, *exit*}, w*=[26,−3,−1] ^5^ and binary reward features ϕ= [*dropped off package at destination*, *entered mud*, *action taken*]. The demonstration in the left image of Figure 1 corresponds to the constraints in the right image. With a unit cost for each action, the constraints on viable reward weights intuitively indicate that 1) ${w\text{*}}_{0}\geq 10$ since a total of 10 actions were taken in the demonstration and that 2) $w_{1}\text{*}\leq -2$ as the detour around the mud took two actions.

### 3.4 Set Cover Optimal Teaching (SCOT)

SCOT (Brown and Niekum, 2019) allows a robot to select the minimum number of demonstrations that results in the smallest BEC area (i.e. the intersection of the constraints) for an IRL learner. As it only considers IRL, it serves as a baseline method to the techniques proposed in this work that augment SCOT with human teaching strategies.

The SCOT algorithm is summarized here for completeness. The robot first translates all possible demonstrations of its policy in a domain into a corresponding set of BEC constraints. After taking a union of these constraints, redundant constraints are removed using linear programming (Paulraj and Sumathi, 2010). These non-redundant constraints together form the minimal representation of BEC(π*). SCOT now iteratively runs through all possible demonstrations again and greedily adds to the teaching set D the demonstration that covers as many of the remaining constraints in BEC(π*), until all constraints are covered.^6^ These steps correspond to lines 2–13 in Algorithm 1.

## 4 Proposed Techniques for Teaching Humans

### 4.1 Scaffolding

The SCOT algorithm efficiently selects the minimum number of demonstrations that results in the smallest BEC area for a pure IRL learner (Brown and Niekum, 2019). Such a learner is assumed to fully grasp these few highly nuanced examples that delicately straddle decision-making boundaries and find any other demonstrations redundant. However, *we posit that the BEC area of a demonstration not only inversely corresponds to the amount of information it contains about the possible values of* w* *, but also inversely corresponds to the effort required for a human to extract that information.* Thus humans will likely benefit from additional scaffolded examples that ease them in and incrementally relax the degrees of freedom of the learning problem.

We develop a scaffolding method for a learner without any prior knowledge, outlined as follows. First, obtain the SCOT demonstrations that contains the maximum information on w*. If space remains in the teaching budget *n* for additional demonstrations, begin scaffolding by sorting all possible demonstrations in a domain according to their BEC areas. Then cluster them using k-means into twice as many clusters as the remaining budget to ensure that no two consecutive demonstrations are nearly identical in BEC area (see Figure 2). Randomly draw *m* candidate demonstrations from every other cluster. Finally from these *n* pools of candidate demonstrations, select the ones that best optimize visuals for the teaching set D (as described in the next section). See lines 16–21 in Algorithm 1. In this paper, the algorithm always divided the BEC areas into 6 clusters, considering every other cluster to correspond to “low”, “medium”, and “high” information respectively.

### 4.2 Simplicity and Pattern Discovery

Though the BEC area of a demonstration provides an unbiased, quantitative measure of the information transferred to a pure IRL learner, *human learners are likely also influenced by the medium of the demonstration, e.g. visuals, and the simplicity and patterns it affords*. For example, visible differences between sequential demonstrations can highlight relevant aspects, while visual clutter that does not actually influence the robot’s behavior (e.g. extraneous mud not in the path of the delivery robot) may distract or even mislead the human.

We perform a greedy sequential optimization for pattern discovery and then for simplicity. We first encourage pattern matching by considering candidates from different BEC clusters (which often exhibit qualitatively different behaviors) that are most visually similar to the previous demonstration.^7^ The aim is to highlight a change in environment (e.g. a new mud patch) that caused the change in behavior (e.g. robot takes a detour) while keeping all other elements constant. We then optimize for simplicity. A measure of visual simplicity is manually defined for each domain (e.g. the number of mud patches in the delivery domain), and out of the scaffolding candidates, the visually simplest demonstration is selected.

The proposed methods for scaffolding and visual optimization come together in Algorithm 1.^8^ Since the highest information SCOT demonstrations are selected first then demonstrations are selected via k-means clustering from high to low information, the algorithm concludes by reversing the demonstration list to order the demonstrations from easiest to hardest (line 28).^9^ $\hat{N}\left[\cdot \right]$ denotes the operation of extracting unit normal vectors corresponding to a set of half-space constraints, and ∖ denotes set subtraction. An example of a sequence of demonstrations that exhibits scaffolding, simplicity, and pattern discovery can be found at the top of Figure 2.

### 4.3 Testing

An optimal trajectory’s BEC area intuitively captures its informativeness as a teaching demonstration. The smaller the area, the less uncertainty there is regarding the value of w*.

We propose a complementary and novel idea: *that the BEC area can be inverted as a measure of a trajectory’s difficulty as a question during testing*, i.e. when a human is asked to predict the robot’s trajectory in a new situation. Intuitively, a large BEC area indicates that there are many viable reward weights for a demonstration, and thus the human does not need to precisely understand w* to correctly predict the robot’s trajectory. We can also use this measure to scaffold tests of varying difficulties to gauge the human’s understanding of w* and subsequently π*.

Algorithm 1 Machine Teaching for Human Learners.

## 5 User Studies

We ran two online user studies that involved participants watching demonstrations of a 2D agent’s policy and predicting the optimal trajectory in new test environments.^10^ The studies were designed to evaluate the following hypotheses.

**H1**: The BEC area of a demonstration correlates 1) inversely to the expected difficulty for a human to correctly predict it during testing, and 2) directly to their confidence in that prediction.

**H2**: The BEC area of a demonstration also correlates 1) inversely to the information transferred to a human during teaching and 2) inversely to the subsequent test performance.

**H3**: Forward scaffolding (demonstrations shown in increasing difficulty) will result in better qualitative assessments of the teaching set and better participant test performance over no scaffolding (only high difficulty demonstrations shown) and backward scaffolding (demonstrations shown in decreasing difficulty), in that order.

**H4**: Positive visual optimization will result in better qualitative assessments of the teaching set and better test performance over negative visual optimization (with positive and negative visual optimization corresponding to the maximization and minimization, respectively, of both simplicity and pattern discovery).

The two user studies jointly tested H1. The first study tested H2 and the second study tested H3 and H4.

### 5.1 Domains

**FIGURE 3:**
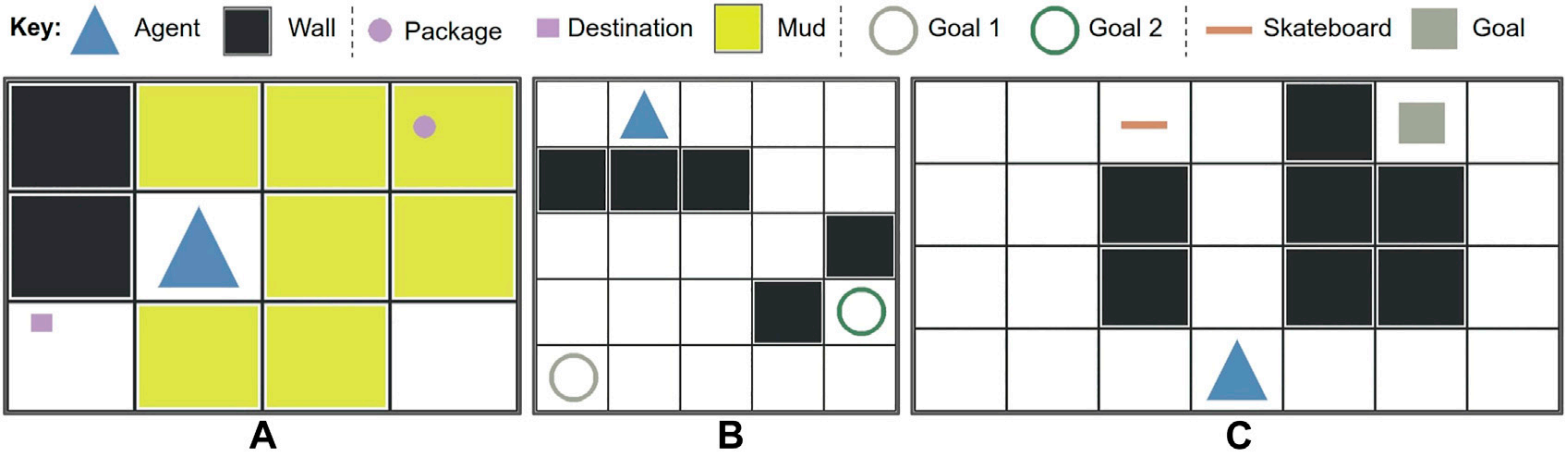
Three domains were presented in the user study, each with a different set of reward weights to infer from demonstrations using inverse reinforcement learning. **(A)** delivery, **(B)** two-goal, **C**: skateboard.

Three simple gridworld domains were designed for this study (see Figure 3). The available actions were {*up*, *down*, *left*, *right*, *pick up*, *drop*, *exit*}. Each domain consisted of one shared reward feature of unit action cost, and two unique reward features as follows.

**Delivery domain**: The agent is rewarded for bringing a package to the destination and penalized for moving into mud.

**Two-goal domain**: The agent is rewarded for reaching one of two goals, with each goal having a different reward.

**Skateboard domain**: The agent is rewarded for reaching the goal. It is penalized less per action if it has picked up a skateboard (i.e. riding a skateboard is less costly than walking).

To convey an upper bound on the positive reward weight, the agent exited from the game immediately if it encountered an environment where working toward the positive reward would yield a lower overall reward (e.g. too much mud along its path). The semantics of each domain were masked with basic geometric shapes and colors to prevent biasing human learners with priors. All domains were implemented using the simple_rl framework (Abel, 2019).

### 5.2 Study Design

The first and second user studies (US1 and US2, respectively) used the same domains, procedures, and measures, though they differed in which variable was manipulated.

US1 explored how BEC area of demonstrations correlates with a human’s understanding of the underlying policy. Thus, the between-subjects variable was *information class*, with three levels: low, medium, and maximum (i.e. SCOT). The low and medium information demonstrations were selected from the fifth and third BEC clusters respectively (see Figure 2). When selecting multiple demonstrations from a *single* cluster, we optimized for visual simplicity and *dissimilarity* as diversity^11^ of demonstrations has been shown to improve human learning (Amir and Amir, 2018; Huang et al., 2019). The number of demonstrations shown in each domain was set to equal the number of SCOT demonstrations for fair comparison (2 for delivery and skateboard, 3 for two-goal).

US2 explored how incorporating human learning strategies impacts a human’s understanding of the underlying policy. Specifically, it examined how the presence and direction of scaffolding, and optimization of visuals, would impact the human’s test performance. The between-subjects variables were *scaffolding class* (none, forward, and backward), and *visual optimization* (positive and negative). For scaffolding class, forward scaffolding showed demonstrations according to Algorithm 1, backward scaffolding showed forward scaffolding’s demonstrations in reverse, and no scaffolding showed all high informative examples from the 1st BEC cluster (Figure 2). Five demonstrations were shown for each domain, always ending with demonstrations determined by SCOT.

Both US1 and US2 had two additional within-subject variables: *domain* (delivery, two-goal, and skateboard, described in Section 5.1) and *test difficulty* (low, medium, and high, determined by the BEC area of the test).

For both user studies, participants first completed a series of tutorials that introduced them to the mechanics of the domains they would encounter. In the tutorials, participants learned that the agent would be rewarded or penalized according to key events (i.e. reward features) specific to each domain. They were then asked to generate a few predetermined trajectories in a practice domain with a live reward counter to familiarize themselves with the keyboard controls and a practice reward function. Finally, participants entered the main user study and completed a single trial in each of the delivery, two-goal, and skateboard domains. Each trial involved a teaching portion and a test portion. In the teaching portion, participants watched videos of optimal trajectories that maximized reward in that domain, then answered subjective questions about the demonstrations (M2-M4, see Section 5.3). In the subsequent test portion, participants were given six new test environments and asked to provide the optimal trajectory. The tests always included two low, two medium, and two high difficulty environments shown in random order. For each of the tests, participants also provided their confidence in their response (M5). The teaching videos for each condition were pulled from a filtered pool of 3 exemplary sets of demonstrations proposed by Algorithm 1 to control for bias in the results. The tests were likewise pulled from a filtered pool of 3 exemplary sets of demonstrations for each of the low, medium, and high difficulty test conditions.

Finally, though the methods described in this paper are designed for a human with no prior knowledge regarding any of the weights, the agent in our user studies assumed that the human was aware of the step cost and only needed to learn the relationship between the remaining two weights in each domain. This simplified the problem at the expense of a less accurate human model and measure of a demonstration’s informativeness via BEC area. However, the effect was likely mitigated in part by the clustering and sampling in Algorithm 1, which only makes use of coarse BEC areas.

### 5.3 Measures

The following objective and subjective measures were recorded to evaluate the aforementioned hypotheses.

**M1**. **Optimal response:** For each test, whether the participant’s trajectory received the optimal reward or not was recorded.

**M2**. **Informativeness rating:** 5-point Likert scale with prompt “How informative were these demonstrations in understanding how to score well in this game?”

**M3**. **Mental effort rating:** 5-point Likert scale with prompt “How much mental effort was required to process these demonstrations?”

**M4**. **Puzzlement rating:** 5-point Likert scale with prompt “How puzzled were you by these demonstrations?”

**M5**. **Confidence rating:** 5-point Likert scale with prompt “How confident are you that you obtained the optimal score?”

## 6 Results

One hundred and sixty two participants were recruited using Prolific (Palan and Schitter, 2018) for the two user studies. Participants’ ages ranged from 18 to 57 (M = 26.07, SD = 8.35). Participants self-reported gender (roughly 67% male, 30% female, 2% non-binary, and 1% preferred to not disclose). Each of the nine possible between-subjects conditions across the two user studies were randomly assigned 18 participants (such that US1 and US2 contained 54 and 108 participants respectively), and the order of the domains presented to each participant was counterbalanced.

The three domains were designed to vary in the difficulty of their respective optimal trajectories. We calculated an intraclass coefficient (ICC) based on a mean-rating (k = 3), consistency-based, 2-way mixed effects model (Koo and Li, 2016) to evaluate the consistency of each participant’s performance across domains. A low ICC value of 0.37 (p<.001) indicated that performance in fact varied considerably across domains for each participant. We subsequently average each participant’s scores across the domains in all following analyses, potentially yielding results that are representative of domains with a range of difficulties.

**H1:** We combine the test responses from both user studies as they shared the same pool of tests. A one-way repeated measures ANOVA revealed a statistically significant difference in the percentage of optimal responses (M1) across test difficulty (F(2,322)=275.35,p<.001). Post-hoc pairwise Tukey analyses further revealed significant differences between each of the three groups, with the percentage of optimal responses dropping from low (M=0.89), to medium (M=0.68), to high (M=0.36) test difficulties (p<.001 in all cases).

**FIGURE 4:**
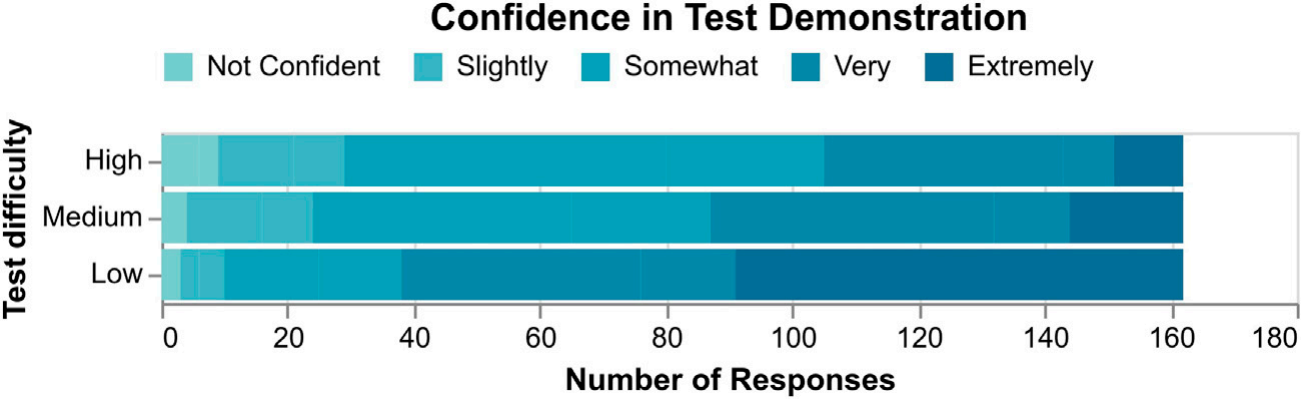
Participants were significantly more confident of their responses as test difficulty decreased.

Spearman’s rank-order correlation further showed a significant inverse correlation between test difficulty and confidence (M5, $r_{s}=-.40,p<.001,N=486$). See Figure 4 for the raw confidence data.

*Objective and subjective results both support H1, that BEC area can indeed be used as a measure of difficulty for testing.* We thus proceed with the rest of the analyses with “test difficulty” as a validated independent variable.

**H2:** A two-way mixed ANOVA on percentage of optimal responses (M1) did not reveal a significant effect of information class of the teaching set (F(2,51)=1.23,p=.30), though test difficulty had a significant effect consistent with the H1 analysis (F(2,102)=118.58,p<.001). There was no interaction between information class and test difficulty (F(4,102)=0.67,p=.61).

**FIGURE 5:**
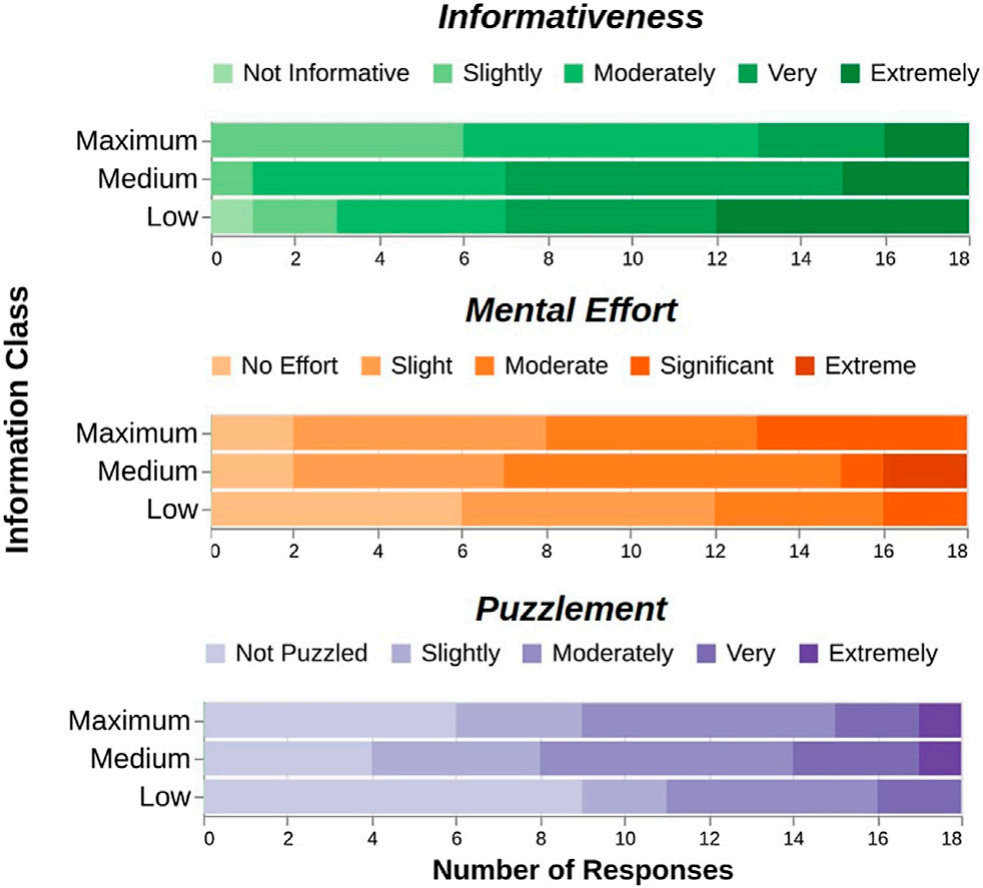
The information class of demonstrations only significantly influences their perceived informativeness, ironically decreasing from low to maximum information class. This suggests that a demonstration’s intrinsic information content (as measured by its BEC area) does not always correlate with the information transferred to human learners. No significant effects were found between information class and mental effort or puzzlement.

Spearman’s correlation test only found a significant negative correlation between information class and perceived informativeness (M2, $r_{s}=-0.28,p=.04,N=54$). Neither mental effort (M3, p=.08) nor puzzlement (M4, p=.36) were found to have significant correlations with information class. See Figure 5 for the raw subjective ratings.

*The data failed to support H2.* The data suggests that IRL alone is indeed an imperfect model of human learning, motivating the use of human teaching techniques to better accommodate human learners.

There was no correlation between information class and test performance, likely a result of two factors. First, the number of demonstrations provided (two or three) across the conditions in US1 were likely too few for human learners, who are not pure IRL learners and can sometimes benefit from “redundant” examples that reinforce a concept. Second, as will be discussed under the scaffolding subsection in Section 7.2, BEC area is likely an insufficient model of a demonstration’s informativeness to a human and warrants further iteration.

Accordingly, maximum information demonstrations provided by SCOT (M=0.61) failed to significantly improve the percentage of optimal responses compared to medium (M=0.65) and low (M=0.67) information demonstrations as IRL would have predicted. The subjective results further indicate that people ironically found the maximally informative demonstrations least informative. We hypothesize that participants struggled to digest the information contained within SCOT’s demonstrations all at once, motivating the use of scaffolding to stage learning into mangeable segments.

**FIGURE 6:**
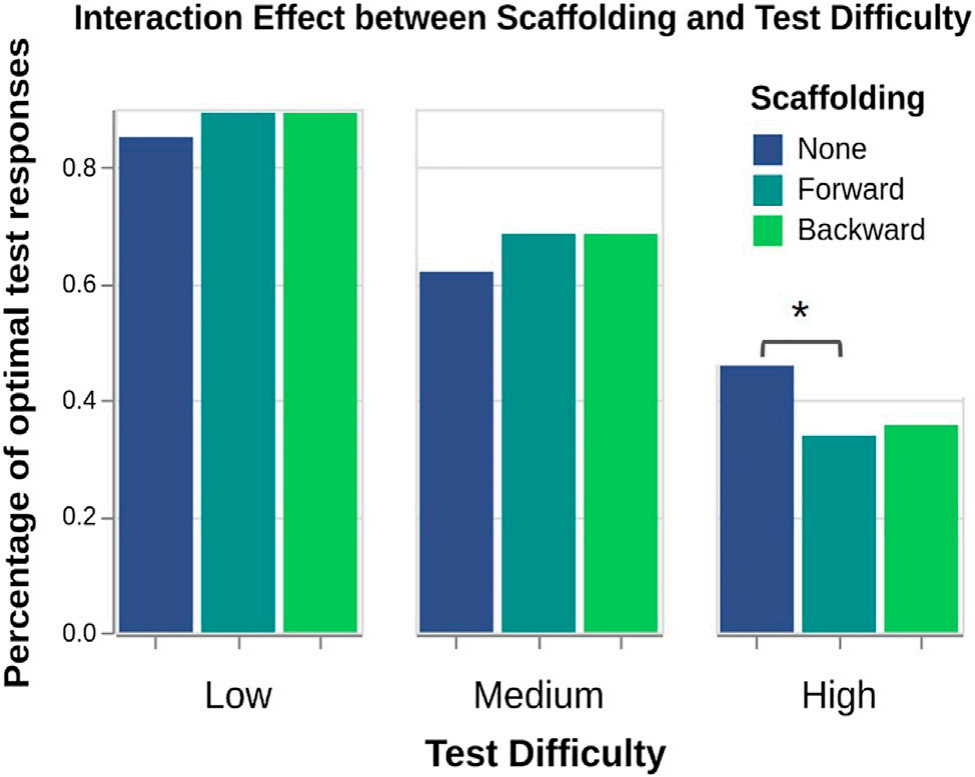
Though the three scaffolding conditions perform similarly in aggregate across all tests, “no scaffolding” significantly increases performance for high difficulty tests.

**H3:** A two-way mixed ANOVA on percentage of optimal responses (M1) revealed a significant interaction effect between scaffolding and test difficulty (F(4,210)=2.79,p=.03). Tukey analyses showed that no scaffolding (M=0.46) yielded significantly better test performance than forward scaffolding (M=0.34) for high difficulty tests (p=.05). Though not statistically significant, a trend of forward and backward scaffolding outperforming no scaffolding on low (M=0.89,0.89,0.85 respectively) and medium difficulty tests (M=0.69,0.69,0.62 respectively) can be observed as well (see Figure 6).

A two-way mixed ANOVA surprisingly did not reveal a significant effect from scaffolding (F(2,105)=0.02,p=.98) but did find a significant effect for test difficulty (F(2,210)=167.63,p<.001) on percentage of optimal responses (M1) as expected.

A Kruskal–Wallis test did not find differences between the informativeness (H(2)=5.18,p=.07), mental effort (H(2)=1.16,p=.56), or puzzlement (H(2)=0.59,p=.74) ratings (M2–M4) of differently scaffolded teaching sets.

*The data largely failed to support H3.* Forward and backward scaffolding surprisingly led to nearly identical test performance. Though no scaffolding performed similarly overall, it yielded a significant increase in performance specifically for high difficulty tests. These two surprising results are addressed in the discussion. The subjective measures did not indicate any clear relationships.

**FIGURE 7:**
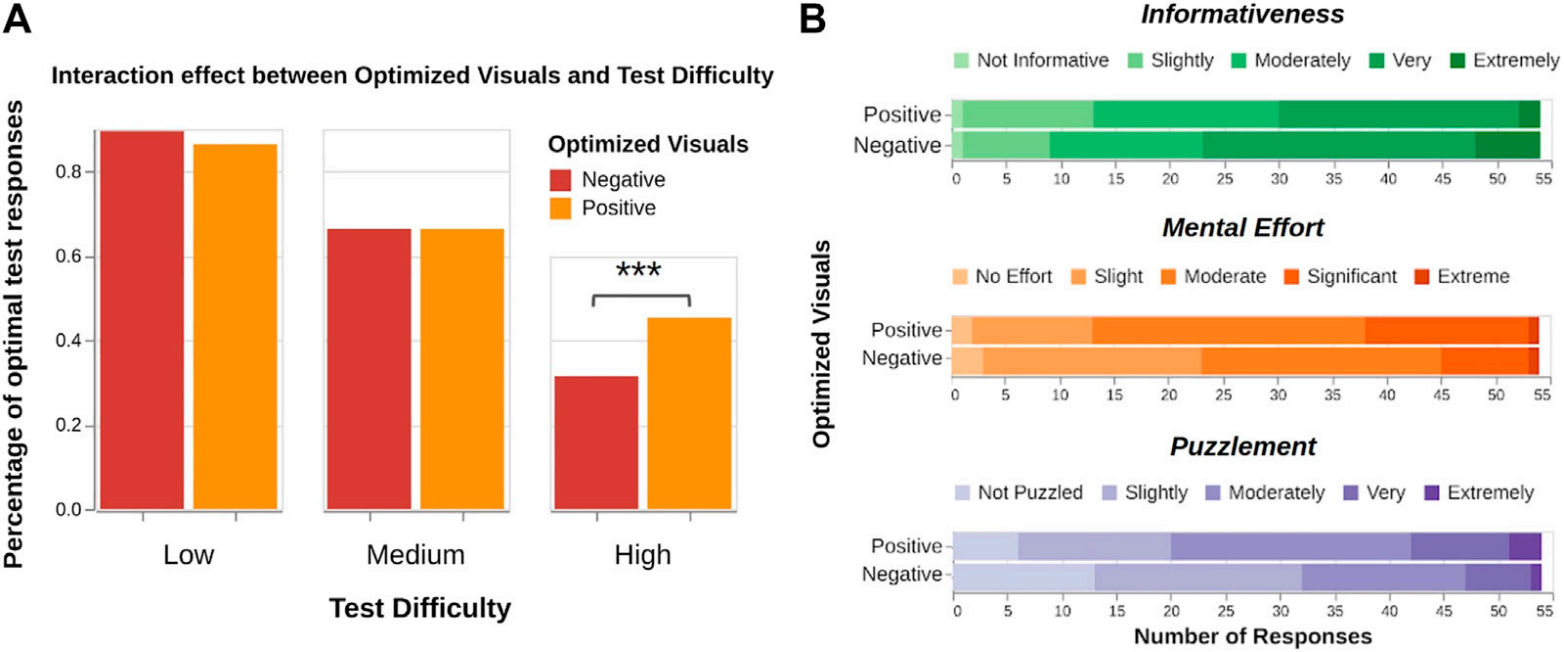
**(A)** Optimizing teaching demonstration visuals does not significant affect performance on low and medium difficulty tests, but leads to a significant improvement on high difficulty tests. **(B)** Ratings on mental effort and puzzlement surprisingly increased for positive visual optimization, likely an artifact of unforeseen study design effects. No significant effects were found for ratings on informativeness.

**H4**: A two-way mixed ANOVA on percentage of optimal responses (M1) revealed significant effects of test difficulty (F(2,212)=169.21,p<.001) and an interaction effect between optimized visuals and test difficulty (F(2,212)=5.61,p=.004). Exploring the interaction effect with Tukey analyses revealed that visual optimization had no effect on test performance on low (p=.24) and medium (p=.90) difficulty tests, but led to a significant improvement in performance in high (p<.001) difficulty tests for positive visual optimization (M=0.45) over negative (M=0.31), see Figure 7. The two-way mixed ANOVA did not reveal a significant from optimized visuals alone (F(1,106)=2.27,p=.13).

A Mann–Whitney *U* test surprisingly found that ratings for mental effort ($U\left(N_{neg}=54,N_{pos}=54\right)=1131.5,p=.03$) and puzzlement ($U\left(N_{neg}=54,N_{pos}=54\right)=1082.5,p=.02$) (M3 and M4) increased for positive visual optimization. Informativeness ratings were not found to differ significantly between the two visual optimizations (p=.11).

*The data partially supports H4.* Optimizing visuals improved test performance for high difficulty tests. However, optimizing visuals also yielded counterintuitive results for the subjective measures on mental effort and puzzlement, which we address in the following section.

## 7 Discussion

### 7.1 Learning Styles

Analyzing the free-form comments provided by participants throughout the user studies revealed unexpected insights about their learning styles. Though this paper assumed that participant learning would only resemble IRL, we discovered it sometimes resembled imitation learning^12^, which models humans as learning the optimal behavior directly from demonstrations (as opposed to through an intermediate reward function like IRL) (Daw et al., 2005; Lage et al., 2019). For example, one participant expounded upon their mental effort Likert rating (M3) with following description of IRL-style learning: “You need to make a moderate amount of mental effort to understand all the rules and outweight [sic] everything and see what is worth it or not in the game.” In contrast, another expounded upon their used mental effort rating with the following description of IL-style learning: “The primary ‘mental effort’ was in memorizing the patterns of each level/stage and matching the optimal movements for them.”

To better understand the types of learning employed by our participants, we analyzed their optional responses to the following questions: “Feel free to explain any of your selections above if you wish:” (asked in conjunction with prompts for ratings of informativeness, mental effort, and puzzlement of demonstrations in each domain, i.e. up to three times) and “Do you have any comments or feedback on the study?” (asked after the completion of the full study, i.e. once). Similar to Lage et al. (2019), we coded relevant responses from participants regarding their thought process as resembling IRL (e.g. “So, the yellow squares should be avoided if possible and they possibly remove two points when crossed but I’m not sure”) or as resembling IL (e.g. “I did not understand the rule regarding yellow tiles. It seems they should be avoided, but not always. Interesting…”), or as “unclear” (e.g. “After some examples I feel like I’m understanding way better these puzzles.”). A second coder uninvolved in the study independently labeled the same set of responses, assigning the same label to 79% of the responses. A Cohen’s kappa of 0.64 between the two sets of codings further indicates moderate to substantial agreement (Landis and Koch, 1977; Altman, 1990; McHugh, 2012). Please refer to the Supplementary Material for the responses, labels, and further details on the coding process.

As Table 1 conveys, both coders agreed that more responses resembled IRL than IL and “unclear” combined, suggesting that people perhaps employed IRL more often than not. However, we note that the way the tutorials introduced the domains may have influenced this result. For example, explicitly conveying each domain’s unique reward features and clarifying that a trajectory’s reward is determined by a weighting over those features may have encouraged participants to first infer the reward weights from optimal demonstrations (e.g. through IRL) and then infer the optimal policy (as opposed to directly inferring the optimal policy e.g. through IL).

**TABLE 1:** Coding of qualitative participant responses as resembling inverse reinforcement learning (IRL) or imitation learning (IL), or “unclear.”

Learning style	Raw counts (across user studies)		Percentages (across coders)	
	Coder 1	Coder 2	User study 1 (%)	User study 2 (%)
IRL	25	27	32	68
IL	7	9	27	12
Unclear	15	11	41	20

Examining the percentage of each response across the two user studies reveals another interesting trend. Responses were far more likely to be coded as IRL in US2, where participants got to see five demonstrations as opposed to US1, where participants only got to see two or three demonstrations. This echoes the observation of Lage et al. (2019) that people may be more inclined to use IL over IRL in less familiar situations, which may be moderated in future studies through more extensive pre-study practice and/or additional informative demonstrations that better familiarize the participant to the domains.

Finally, out of 15 participants who provided more than one response, coders agreed that eight appeared to employ the same learning style throughout the user study (e.g. participants 129 and 142 in US2 only provided responses resembling IRL), four appeared to have changed styles through the user study (e.g. participants 59 in US1 and 20 in US2 provided various responses that resembled IL, IRL, or were unclear), and three were ambiguous (i.e. one coder coded a consistent learning style while the other did not). Though we controlled for learning effects by counterbalancing the order of the domains, participants likely found the domains to vary in the difficulty of their respective optimal trajectories (as suggested by the ICC score). Furthermore, certain conditions led to significant differences in subjective and objective outcomes (e.g. maximum information demonstrations were ironically perceived to be least informative (H2) and positive visual optimization improved performance for high difficulty tests (H4)). We thus hypothesize that the varying difficulties in domains and conditions non-trivially influenced the learning styles at different times [e.g. by moderating familiarity (Lage et al., 2019)].

*Future work:* The multi-faceted nature of human learning can be described by a number of models such as IRL and IL. Lage et al. (2019) show post hoc that tailoring the teaching to the human’s favored learning style can improve the learning outcome. Thus, predicting a human’s current learning style a priori or in situ (e.g. by using features such as the human’s familiarity of the task or domain) and matching the teaching appropriately in real time will be an important direction of future work.

### 7.2 Scaffolding

Though BEC area is a well-motivated preliminary model of a demonstration’s informativeness to a human, backward scaffolding’s unexpected on-par performance with forward scaffolding suggests that it is insufficient and our scaffolding order likely was not clear cut in either direction. In considering possible explanations, we note that Eq. 4 presents a computationally elegant method of generating BEC constraints via sub-optimal, one-step deviations from the optimal trajectory. However, these suboptimal trajectories do not always correspond to the suboptimal trajectories in the human’s mind (e.g. which may allow more than one-step deviations). This sometimes leads to a disconnect between a demonstration’s informativeness as measured by BEC area and its informativeness from the point of view of the human.

Furthermore, forward and backward scaffolding (each comprised of low, medium, and high information demonstrations) yielded higher performance for low and medium difficulty tests, and no scaffolding (comprised of only high information demonstrations) yields significantly higher performance for high difficulty tests. Improved performance when matching the informativeness and difficulty of teaching and testing demonstrations respectively (which yields similar demonstrations) further suggests that IL-style learning may have also been at play.

Finally, participants across each condition never achieved a mean score of greater than 0.5 for high difficulty tests, indicating that they were largely unable to grasp the more subtle aspects of the agent’s optimal behavior. While the five demonstrations shown in US2 should have conveyed the maximum possible information (in an IRL-sense), they were not as effective in reality. One reason may be that human cognition is constrained by limited time and computation (Griffiths, 2020), and at times may opt for approximate, rather than exact, inference (Vul et al., 2014; Huang et al., 2019). Approximate inference (and even IL-style learning) indeed would have struggled with high difficulty tests whose optimal behavior could often only be discerned through exact computation of rewards. In addition to potentially showing more demonstrations (including “redundant” demonstrations that reinforce concepts and are still useful for approximate IRL), we believe that more effective scaffolding that further simplifies the concepts being taught while simultaneously challenging human’s current knowledge will be key to addressing this gap, as we discuss next.

*Future work:* We propose two directions for future work on scaffolding. First, we note that our selected demonstrations often revealed information about multiple reward weights at once, which could be difficult to process. Instead, we can further scaffold by teaching about one weight at a time, when possible. Second, Reiser (2004) suggests that scaffolding should not only provide structure that reduces problem complexity but at times induce cognitive conflict to challenge and engage the learner. The current method of scaffolded teaching assumes that the learner has no prior knowledge when calculating a demonstration’s informativeness (e.g. Algorithm 1 considers a repeat showing of a demonstration to a learner to be equally as informative as the first showing). But when filtering for teaching and testing sets for the user studies, we sometimes observed and accounted for the fact that demonstrations with the same BEC area could further vary in informativeness or difficulty to different learners based on whether it presented an expected behavior or not. We believe that providing demonstrations which incrementally deviate from the human’s current model will be more informative to a human and would be better suited to scaffolding.

### 7.3 Simplicity and Pattern Discovery

Optimizing visuals improved test performance, but only for high difficulty tests. This suggests that simplicity and pattern discovery could produce a meaningful reduction in complexity for only high information demonstrations (which contain the insights necessary to do well on the high difficulty tests), while those of low and medium information were already comprehensible.

We found counterintuitive results on mental effort or puzzlement ratings (M3–M4) for H4, where ratings for mental effort and puzzlement increased from negative to positive visual optimizations. One factor may have been the open-ended phrasing of the corresponding Likert prompts that failed to always elicit the intended measure. For example, one participant expounded upon their mental effort rating by saying “it takes a bit of efford [sic] remembering that you can quit at any time,” referencing the difficulty of remembering all available actions rather than the intended difficulty of performing inference over the optimal behavior.

Similarly, the open-ended prompt for puzzlement failed to always query specifically for potential puzzlement arising from (a potentially counterintuitive) ordering of the demonstrations. Instead it sometimes invited comments such as ‘I think i [sic] saw the same distance to the objective 2 times and 2 differnt [sic] outcomes,’ and interestingly informed us of possible unforeseen confounders on puzzlement such as limited memory. As participants were not allowed to rewatch previous demonstrations to enforce scaffolding order, similar demonstrations (in correspondingly similar environments) were sometimes mistaken to have shown different behaviors in the same environment.

*Future work:* Future iterations would benefit from “marking critical features” that “accentuates certain features of the task that are relevant”, as suggested by Wood et al. (1976). For example, imagine showing two side-by-side demonstrations in the delivery domain, one where the robot exits because of the many mud patches in its path and one where the robot completes the delivery because of one fewer mud patch in its path. Outlining the presence and absence of the critical mud patch with a salient border in the two demonstrations respectively would help highlight the relevant cause for the change in robot behavior to the learner.

### 7.4 Testing

Objective and subjective results strongly support BEC area as a measure of test difficulty for human learners. Following studies may thus use tests of varying BEC areas and difficulties to evaluate and track the learner’s understanding throughout the learning process.

*Future work:* Effective scaffolding is contingent on maintaining an accurate model of the learner’s current abilities. Though this work assumed disjoint teaching and testing phases, learning is far more dynamic in reality. Future work should therefore explore how to select an initial set of tests that can accurately discern the learner’s current knowledge, and also to know when to switch between teaching and testing throughout the learning process.

### 7.5 Real-world Applicability

Though the proposed method of machine teaching is theoretically general, there are additional considerations that must be addressed for real-world applicability.

First, a robot’s policy may be a function of many parameters. Though performing IRL in a high-dimensional space may sometimes be warranted, humans naturally exhibit a bias toward simpler explanations with fewer causes (Lombrozo, 2016) and can only effectively reason about a few variables at once (e.g. Halford et al. (2005) suggest the limit to be around four). Thus, future work may examine approximating a high-dimensional policy with a low-dimensional policy that can be conveyed instead with minimal loss. Additionally, scaffolding methods that explicitly convey only a subset of the reward weights at a time should be developed as previously noted.

Second, a robot’s entire trajectory will not always be necessary or reasonable to convey if it is lengthy. Thus techniques that extract and convey only the informative segments along with sufficient context will be important. For segments that are infeasible to convey in the real world (e.g due to necessary preconditions not being met), demonstrations may be given in simulation instead.

## 8 Conclusion

As robots continue to gain useful skills, their ability to teach them to humans will benefit those looking to acquire said skills and also facilitate fluent collaboration with humans. In this work, we thus explored how a robot may teach by providing demonstrations of its skill that are tailored for human learning.

We augmented the common model of humans as inverse reinforcement learners with insights from learning theory and cognitive science to better accommodate human learning. Scaffolding provided demonstrations that increase in informativeness and difficulty, aiming to ease the learner into the skill being taught. Furthermore, simple demonstrations that conveyed a discernible pattern were favored to minimize potentially misleading distractions and instead highlight critical features. Finally, a measure for quantifying the difficulty of tests was proposed toward effective evaluation of learning progress.

User studies strongly correlated our measure of test difficulty with human performance and confidence. Favoring simplicity and pattern discovery when selecting teaching demonstrations also led to a significant increase in performance for high difficulty tests. However, scaffolding failed to produce a significant effect on the test performance, informing both the shortcomings of the current implementation and the ways it can be improved in future iterations. Finally, though this work assumed disjoint teaching and testing phases with a static human model, effective scaffolding requires the teacher query, maintain, and leverage a dynamic model of the student to tailor the learning appropriately. We leave this as an exciting direction for future work.

## Data Availability

The code for the human teaching techniques can be found in the following repository: https://github.com/SUCCESS-MURI/machine-teaching-human-IRL. The code for generating the user study (including videos of the teaching and testing demonstrations) and the data corresponding to our results can be found in the following repository: https://github.com/SUCCESS542 MURI/psiturk-machine-teaching.

## Footnotes

1
Ng and Russell (2000) suggest that “the reward function, rather than the policy, is the most succinct, robust, and transferable definition of the task.”

2 Though we assume a deterministic MDP, the methods described here naturally generalize to MDPs with stochastic transition functions and policies.

3 This assumption can be made without loss of generality as the reward features can be nonlinear with respect to states and actions and be arbitrarily complex.

4 In principle, a robot could simply convey w* explicitly to a human. However, it can be nontrivial for humans to map precise numerical reward weights to the corresponding optimal behavior through planning, especially if there is large number of reward features. Thus, providing demonstrations that inherently carry information regarding w* and directly conveying the optimal behavior can be more a effective teaching method for human learners.

5 In practice, we also require that ${\left\|w\right\|}_{1}=1$ to circumvent the scaling invariance of IRL solutions and to eliminate the degenerate all-zero reward function (Brown and Niekum, 2018). We convey the non-normalized w here for intuition.

6 Instead of greedily adding the first demonstration that covers the most remaining constraints of BEC(π*) at each iteration, one can enumerate all possible combinations of demonstrations that cover BEC(π*) and optimize for simplicity and pattern discovery here as well.

7 We measure the visual similarity of two states by defining a hash function over a domain’s state space and calculating the edit distance between the two corresponding state hashes.

8 An implementation is available at https://github.com/SUCCESS-MURI/machine-teaching-human-IRL.

9 In theory, one could order SCOT and k-means demonstrations jointly by BEC area and potentially allowing them to mix in order. However, a SCOT demonstration that contributes a maximally informative constraint of BEC(π*) may in fact have a large BEC area. Thus, showing this SCOT demonstration early on may actually render a later k-means demonstration as uninformative (i.e. the SCOT demonstration’s BEC(π*) constraint may cause a later k-means demonstration’s constraints to be redundant). Instead, showing k-means demonstrations that iteratively decrease in BEC area, then showing SCOT demonstrations ensures that the learner receives non-redundant constraints on w* at each step.

10 Code for the user studies, videos of teaching and testing demonstrations, and the collected data are available at https://github.com/SUCCESS-MURI/psiturk-machine-teaching.

11 Note that Algorithm 1 already achieves diversity by scaffolding demonstrations across *different* BEC clusters and thus benefits instead from visual similarity.

12 Note that the term “behavior cloning” is sometimes used instead to refer to the process of directly learning the optimal behavior. Accordingly, “imitation learning” is sometimes used to refer to the broad class of techniques that learn optimal behavior from demonstrations, encompassing both behavior cloning and IRL (Osa et al., 2018).
