# Supplementary material for: Machine Teaching for Human Inverse Reinforcement Learning
Source: Front Robot AI. 2021 Jun 30;8:693050. doi: 10.3389/frobt.2021.693050 (PMC8278287; doi:10.3389/frobt.2021.693050)
Supplement: Supplementary file 1 [file Table1.pdf]

# Supplementary Material

## 1 QUALITATIVE RESPONSES REGARDING LEARNING STYLE

Participants optionally responded to the following two questions throughout the study: “Feel free to explain any of your selections above if you wish:” (asked in conjunction with prompts for ratings of informativeness, mental effort, and puzzlement of demonstrations in each domain, i.e. up to three times) and “Do you have any comments or feedback on the study?” (asked after the completion of the full study, i.e. once). Thus, each participant could provide up to four responses.

While both questions were open-ended, many participants provided responses that provided insight into how they performed inference over the optimal behavior in new situations. Thus, the lead author pulled a subset of the responses to be coded that either demonstrated an attempt at understanding a specific aspect of a domain’s reward structure (e.g. mud/yellow squares yielding negative reward), deducing the corresponding optimal behavior (e.g. avoid mud/yellow squares if possible), or meta-level comments on the inference performed through out the user study (e.g. seeing the user study as a “guessing game trying to figure out reward values and such...”). Other comments such as rote recalls of particular training demonstrations (which reveals data used to perform inference, but not the inference mechanism itself), imprecise remarks of confusion (perhaps over an aspect of a domain with no allusion to how it may affect the optimal behavior), and overall impressions on user study were not included in the coding set.

Comments in the coding set were independently coded by the lead author and a second coder uninvolved in the study as resembling inverse reinforcement learning (IRL), imitation-learning (IL), or as ‘unclear’. All responses to the two questions (including those unrelated to inference) can be found in the study data that is available in the following repository: https://github.com/SUCCESS-MURI/psiturk-machine-teaching. Please note that references to ‘Chip’ in the responses below are to the agent that behaved optimally in each domain.

Table S1: Coding qualitative participant responses with learning styles (User study 1)

| Participant ID | Coder 1 | Coder 2 | Response |
|---|---|---|---|
| 1 | Unclear | IRL | I’m not sure at this stage why the robot would choose to go to one ring over another. |
| 1 | Unclear | IRL | The study required a certain amount of inference from me rather than following explicit instructions. |
| 34 | Unclear | IRL | I don’t believe this video is as informative as the other ones because I think it should clarify the following situation: if Chip has to move the same amount of ‘houses’ to go to one of the circles, which one would he go to? Because we’ve only seen Chip going into the green circle but is that because the green is ‘better’ than the gray or did it choose the green circle because it was the nearer circle? |
| 35 | IRL | IRL | I’m still not sure how any of these affect point values or such |

*continues on next page*

| | | | |
|---|---|---|---|
| 35 | IRL | IRL | This was a really interesting survey, I like the aspect of it as some sort of a guessing game trying to find out reward values and such, thank you for this opportunity! |
| 37 | Unclear | Unclear | I'm honestly really puzzled by these games, I hope the next page will explain the scoring. |
| 56 | IL | IL | Goal seems to be to get to the grey square and avoid everything else |
| 56 | IL | IRL | Not sure if the goal is the nearest ring or the green ring |
| 59 | IL | IL | The primary 'mental effort' was in memorising the patterns of each level/stage and matching the optimal movements for them. |
| 59 | IRL | IRL | The role of the yellow squares in affecting my score was somewhat confusing in these demonstrations. |
| 81 | IL | Unclear | I did it mostly by intuition after analyzing the puzzles for a brief moment. |
| 81 | Unclear | Unclear | After completing some of the puzzles I realized it was better to probably exit some of them. |
| 81 | Unclear | Unclear | After some examples I feel like I'm understanding way better these puzzles. |
| 98 | Unclear | Unclear | Slowly i [sic] understand the game more and more |
| 98 | Unclear | Unclear | It took me a bit to understand how it works, but as soon i [sic] got it, it was a great game |
| 151 | IL | IL | I think there is no reason to pick up the bar if its [sic] not on the way |
| 151 | IRL | IL | Deliver the circle is priority i [sic] guess |

Table S2: Coding qualitative participant responses with learning styles (User study 2)

| Participant ID | Coder 1 | Coder 2 | Response |
|---|---|---|---|
| 7 | IRL | IRL | I think going to the square earns & moving with the rectangle. I *think* moving without the rectangle loses points... |
| 7 | IRL | IRL | I think the both rings are rewarding (green>grey) but moving is negative. |
| 7 | Unclear | IL | Deliver circle good, yellow squares bad |
| 8 | Unclear | IL | I couldn't understand in which case it was better to pick up the rectangle. |

*continues on next page*

| | | | |
|---|---|---|---|
| 18 | Unclear | Unclear | Confused a bit about which is the best ring to go to in some of these examples |
| 20 | Unclear | Unclear | The videos were moderately informative but did not explain rules at all, so I have to depend on my own interpretation which may, or may not, be correct. But that's the stated purpose I think. |
| 20 | IRL | IRL | I did not truly understood why green circle is preferred (worth more points?) but gray one is acceptable as well sometimes (getting to green would be too costly and getting to gray would make less profit but still better than quitting?) |
| 20 | IL | IL | I did not understand the rule regarding yellow tiles. It seems they should be avoided, but not always. Interesting... |
| 21 | IRL | IRL | not sure whether i [sic] get taxed going into the yellow squares |
| 29 | IRL | IRL | I wonder for one of the instances where the orange rectangle was very few moves away (i.e., 3 or fewer) and would be with Chip for all the remaining moves until reaching the gray square how the game points would play out. Actions taken with the orange rectangle and actions without taking the orange rectangle both affect the score, but I am not sure how (which is positive or negative). |
| 29 | IRL | IRL | Sometimes Chip grabbed the circle in more moves than necessary to retrieve it and bring it back to purple square. For another, when choosing to grab the circle, Chip moved onto white squares instead of yellow squares. Moving into a yellow square would be an action that affects the score, but in what way? This demonstration would imply negatively. |
| 47 | IRL | IRL | I was unsure why chip decided to exit or decided to choose x or y path. It was pretty confusing. In one instance though with the board covered in yellow, I assumed chip would end up with a pretty negative score so chip exited. Overall though, it is confusing. |
| 64 | IRL | IRL | Well the only thing really missing is the amount of points each action does |
| 103 | Unclear | Unclear | I think it's not as informative as the first one, there are a lot more of movements, sometimes it picked the red rectangule [sic] sometimes it didn't, so I'm still trying to think when to pick and when don't pick it. |
| 103 | IL | IL | I'm kind of puzzled, do I have to take one ring to another if possible? |

*continues on next page*

| | | | |
|---|---|---|---|
| 105 | IRL | IRL | I'm not sure what the best strategy is, because I don't don't know the value of the circles |
| 105 | IRL | IRL | I think moving without the orange square take more points but i'm [sic] not sure |
| 129 | IRL | IRL | You need to make a moderate amount of mental effort to understand all the rules and outweight [sic] everything and see what is worth it or not in the game. |
| 129 | IRL | IRL | I think this left me very puzzled because it wasn't easy to differentiate the value of the rings. |
| 129 | IRL | IRL | This required a significant effort to understand the value of the square. |
| 136 | Unclear | IL | JUst [sic] will be hard to understand when to quit or when to pick or not the orange line but i will do my best |
| 142 | IRL | IRL | If I would be able to see the demonstrations back and forth I would eventually get there, not a specific scoreline but within limits. For instance, the last demonstration tells me that if moving is -1, then scoring must be higher than 8 since chip went for it |
| 142 | IRL | IRL | Ok, so this time around I got way better because I didn't get it the first time. Also, this puzzle is easier since there are basically only two variables. Since this is a comparison between green and gray, I did a mathematical system on paint and got the information I needed. I'm still unsure about the exact values but my calculations tell me that if moving is -1, then gray is around +6 and green is around +10. This is all based on the system I've come up with. For example, if Chip would move to green in 8 moves, that tells me that moveValue * 8 + greenValue is positive, and since I'm assuming moving is -1, then this means greenValue - 8 $>$ 0 which means greenValue $>$ 8. |
| 142 | IRL | IRL | a = yellow value, b = white value, c = objective value.<br>1) $a + 8b + c > 0$<br>2) $2a + 4b + c > 0$<br>3) $2a + 6b + c > 0$<br>4) $6a + 2b + c < 0$<br>5) $5a + 5b + c > 0$<br><br>if b = - 1 then<br>1) $a + c - 8 > 0$<br>2) $2a + c - 4 > 0$<br>3) $2a + c - 6 > 0$ |

*continues on next page*

| | | | |
|---|---|---|---|
| | | | 4) 6a + c - 2 < 0<br>5) 5a + c - 5 > 0<br><br>with this equations overlapped this tells me that a <= -3, c >= 20 so moving to yellow is -3, getting objective is 20, assuming white is -1 |
| 145 | IRL | IRL | I was trying to attribute values to the rings but weren't able, just saw that green > grey |
| 147 | Unclear | Unclear | I'm not sure about which ring I should prioritize. |
| 147 | IRL | IRL | So, the yellow squares should be avoided if possible and they possibly remove 2 points when crossed but I'm not sure |
| 154 | IRL | Unclear | I think the green ring is better than the gray ring? |
| 156 | IRL | IRL | I chosen Moderately [sic] informative in first question because I am not sure if there were enough differnent [sic] possibilities shown in demonstrations to assess how many points we get for specififc [sic] action. |
| 157 | IRL | IRL | I think that score system should be explained right away with new "mechanic". Yellow squares made me wonder if they're -2 but I could only guess |
